# Enhancing Natural Rubber Tearing Strength by Mixing Ultra-High Molecular Weight Polyethylene Short Fibers

**DOI:** 10.3390/polym15071768

**Published:** 2023-04-01

**Authors:** Jun He, Baoyuan Huang, Liang Wang, Zunling Cai, Jing Zhang, Jie Feng

**Affiliations:** 1 College of Materials Science and Engineering, Zhejiang University of Technology, Hangzhou 310014, China; 2 Linhai Weixing New Construction Materials Co., Ltd., Taizhou 317016, China

**Keywords:** UHMWPE, short fiber, natural rubber, conveyor belt, tear resistance

## Abstract

Rubber products generally need to have high resistance to abrasion, tear, and cutting. Filling short fiber with strong mechanical properties and forming a net in the rubber matrix is a good method to realize the above aims. In this article, ultra-high molecular weight polyethylene (UHMWPE) short fibers with a diameter of 20 μm and a length of 2 cm were filled into natural rubber (NR) to improve the tear strength of the NR. The influence of the short fiber mass fraction and vulcanization conditions on the mechanical properties of the composites were investigated. The results show that the milling process and vulcanization conditions are key factors in enhancing tear resistance performance. Double-roll milling and vulcanization at 143 °C for 40 min result in strong interfacial adhesion between the UHMWPE short fibers and the NR. The addition of 2 phr of UHMWPE fiber increases the tear strength of the composite material by up to 150.2% (from 17.1 kN/m to 42.8 kN/m) while also providing excellent comprehensive performance. Scanning electron microscope (SEM) imaging confirmed that the UHMWPE short fibers are dispersed in the NR matrix homogeneously, and the interface is close and compact. As a control experiment, UHMWPE resin powder was directly filled into the NR, and then the composite was vulcanized using the same process as that used for the NR/UHMWPE short fiber composite. The results show that the mechanical strength of the NR/resin powder composite exhibits minor improvement compared with NR. As there is no complicated surface modification of the UHMWPE fiber, the results reported may be helpful in improving the tear resistance of the industrially prepared rubber conveyor belts.

## 1. Introduction

Rubber conveyor belts are some of the most important industrial transportation tools in use today, and so are required to have high strength, flame retardancy, and high resistance to abrasion, high temperature, fatigue, tear, and impact [1]. Among these properties, due to severe working conditions, the conveyor belt especially needs to have tear resistance [2] and impact resistance [3]. Recently, differing from the usual inorganic fillers that are employed, the use of fibers for reinforcing rubber has shown great potential. In the last decades, short fiber/rubber composites (SFRC) have been studied in combination with various types of fibers due to their superior physical and chemical properties [4], such as glass fiber [5], carbon fiber [6], ultra-high molecular weight polyethylene (UHMWPE) fiber [7], aramid fiber [8], and natural fiber [9,10,11].

Among all the types of short fibers, carbon fiber, UHMWPE fiber, and aramid fiber may be the three most well-used fibers for improving the mechanical performance of rubber. The modulus of carbon fiber is far higher than that of rubber, so its reinforcing effect is not particularly high [12], although there is good compatibility between the two substances. Aramid fiber is a polar fiber, and so has poor compatibility with most non-polar rubbers [13]. On the contrary, the modulus of UHMWPE fiber is well-matched with non-polar rubbers and exhibits good intrinsic compatibility. Moreover, it also has a low density, high tensile strength, and high resistance to abrasion and thus is expected to exhibit good performance in rubbers [14]. 

The effect of filling untreated or chromic-acid-treated UHMWPE short fibers (the length is 0.5 cm) on natural rubber (NR) was investigated by Li et al. [15]. The tear strength of the fiber/NMR composite consisting of untreated or treated fiber increased no more than 30% compared with that of NR alone. Zhang et al. [16] treated UHMWPE fibers with ozone and then UV grafting glycidyl methacrylate (GMA) in order to enhance the interfacial properties of the fiber/rubber composites. The result showed that by adding an amino-containing adhesive RA reagent to the formula, the adhesive force between the fibers and SBR increased by 79% over that of the untreated fibers. However, in their study, no tensile and tear strengths for the fiber/rubber composites were reported. Tu et al. [17] constructed a polydopamine (PDA) functionalization platform and then deposited zinc oxide nanoparticles on the UHMWPE fiber surface and found that the interfacial adhesion with the rubber matrix was enhanced by 85.4%. Later, they [18] proposed a lower-cost surface modification strategy by replacing expensive dopamine with the catechol/tetraethylenepentamine two-component system. 

Although further surface modification to the UHMWPE fiber has provided better interfacial performance between the fibers and the non-polar rubber, these modifications are complicated and may not be cost-effective [19,20]. In fact, the UHMWPE fibers have good compatibility with most non-polar rubbers because they have similar chemical groups. Further surface modification to the UHMWPE fiber may be unnecessary except for the simple formation of a covalent bond at the interface. On the contrary, the length of the UHMWPE short fiber and the vulcanization process may be more beneficial in increasing the tear strength and other mechanical properties of the rubber. 

In the practical production of rubber products such as jugged triangular belts, aramid short fibers can be filled into the rubber in order to avoid stress cracking at the bottom of such structures. The length of such aramid short fibers lies in the region of 1~3 mm and can often be shorter than 1 mm. However, the cost of the aramid short fibers is particularly expensive (250–400 RMB per kg), and so few industrial-scale plants are willing to use them. Compared with aramid short fiber, UHMWPE short fiber is cost-effective (150–250 RMB/kg). If UHMWPE could be homogeneously dispersed in the rubber matrix, a reinforcing effect may be obtained, similar to the function provided by the aramid short fibers. Additionally, fibers that are longer in length will help improve the tear strength of the resulting composite more obviously. 

Based on promoting the practical application of the UHMWPE short fibers in the rubber industry, in this work, complex and expensive physical or chemical modifications to the fiber surface have neither been used. On the contrary, different amounts of original UHMWPE short fibers with lengths of 2 cm were homogeneously dispersed in NR by double-roll milling at a certain temperature. The mixture was then vulcanized at a specific temperature. The effects of the fiber amount, the mixing, and the vulcanization process on the mechanical performance of the rubber were studied systemically. The results showed that under vulcanization conditions of 143 °C for 40 min, The tearing performance of the NR is increased by up to 150.2% following the addition of 2 phr of UHMWPE fiber. This preparation method does not require any surface modification of the UHMWPE fiber; thus, the results of this study may be beneficial to the rubber conveyor belt industry. 

## 2. Experimental Section

### 2.1. Materials

The NR and other chemical reagents (industrial grade) were obtained from Zhejiang Fenfei Rubber & Plastic Products Co., Ltd. (Taizhou, Zhejiang, China). The UHMWPE short fibers were purchased from Zhejiang Qianxilong Special Fiber Co., Ltd. (Jinhua, Zhejiang, China). The diameter and the length of the short fibers were approximately 20 μm and 2 cm, respectively. The master batch of the NR was prepared by mixing NR, carbon black (CB), aromatic oil (oil), zinc oxide (ZnO), stearic acid (SA), sulfur, accelerator (TBBS), and UHMWPE short fibers. The specific formula of the composite is listed in Table 1. 

### 2.2. Preparation of NR/UHMWPE Short Fiber Composites

The components listed in Table 1 were dried in an electric thermostatic drying oven at 60 °C for 12 h and weighed according to the formulation. The NR and short fibers were mixed on a two-roll mill (LRM-S-150/3E, Labtech Engineering Co., Ltd., Bangkok, Thailand) over a three-step process. First, rubber was mixed with CB, aromatic oil, ZnO, and SA at 80 °C with a speed of 15/12 rpm (front/rear roll). After 3 min, the UHMWPE short fibers were added and milled for a further 10 min. The shear force generated by the rolls leads to most short fibers being oriented in the rolling direction, as reported by Andideh M [21]. Finally, the NR/fiber composites were obtained after sulfur and accelerant (TBBS) were incorporated into the system by mixing for another 3 min. In the two later steps, the milling condition is the same as that of the first step. 

Due to the macroscopic length of the UHMWPE short fiber (2 cm), the oriented fibers presenting on the composite sample surface can be seen clearly. In addition to using a two-roll milling process, a closed mixer was also applied in order to mix the components listed in Table 1 for future industrial manufacture. The temperature was 105 °C, the rotor speed was set at 22 rpm, and the mixing time was 10 min. The fibers were mixed homogeneously into the NR using this process. However, the composites that were mixed with the closed mixer still needed to be formed into sample pieces by two-roll milling. During the two-roll milling process, the fibers were again orientated along the direction of the roll rotation. Thus, for most samples in this study, the fibers were only mixed into NR via the two-roll milling process. 

The creep and melting temperature of the UHMWPE short fibers are close to the vulcanization temperature of general rubber. Therefore, in order to determine the optimized vulcanization temperature, the fiber/NR composite sheets with 2 mm thickness were firstly vulcanized under a thermal pressing machine (LP-S-50, Labtech Engineering Co., Ltd., Bangkok, Thailand) at 140 °C for 40 min, 143 °C for 40 min and 150 °C for 30 min, respectively. Additionally, all the specimens were stored for 24 h at room temperature before further testing. Next, the samples consisting of different fiber content were studied based on the optimal vulcanization process. To further demonstrate the function of the UHMWPE short fiber, UHMWPE resin powder (Shanghai Lianle Chemical Co., Ltd., Shanghai, China, mesh number 100) was directly filled into the NR, and the composites were vulcanized using the same process as used for the NR/short fiber composite. 

### 2.3. Measurements and Characterization

#### 2.3.1. Thermal Property of the UHMWPE Short Fiber

Irrespective of using milling or vulcanization with NR, the thermal performance of the UHMWPE short fiber should be investigated; otherwise, their excellent mechanical properties would disappear as soon as the temperature exceeds the fiber’s creep or melting temperatures. Thus, differential scanning calorimetry (DSC, Mettler Toledo, Zurich, Switzerland) was used to characterize the thermal behavior of the fiber under a nitrogen atmosphere and heated at 10 °C/min from room temperature to 200 °C. 

#### 2.3.2. Curing Characteristics of the Fiber/NR Composite

In order to ensure a suitable vulcanization time for the composite, the NR samples without the UHMWPE short fiber filling were vulcanized at 143 °C and 150 °C by a rheometer (M-3000A, Gotech testing machines Inc., Taiwan, China) until the T90 was measured. The curing characteristic data of the fiber/NR composites were determined using the same rheometer at a temperature of 143 °C. The pressure for vulcanization was 7.5 MPa.

#### 2.3.3. Mechanical Properties of the Fiber/NR Composite

The tensile properties and tear strength of the cured fiber/NR composite samples were determined by an electronic universal testing machine (5966, instron, Boston, MA, USA) based on ISO 37-2005 (Type 2) and ISO 34-1-2010 (trouser test piece), respectively. For the conveyor belt, horizontal tearing is more problematic, especially longitudinal tearing (along the running direction). The test was performed perpendicular to the orientation of the fiber. A Shore hardness tester (LX-A, Shanghai Precision Instruments Co., Ltd., Shanghai, China) was used to measure the hardness under ASTM-D2240 conditions. In each test, 5 replicas of specimens were used, and the average and standard deviation of each test was given in the form of a table. 

The difference inelasticity difference between the fiber and the NR resulted in disconnection when the composite was stretched too long; the application of UHMWPE resin powder was expected to result in the formation of shorter fibers in situ during the filling process. NR, UHMWPE resin powder, and additives were mixed using the same processing as used for the preparation of the NR/UHMWPE short fiber composite. Further, 2 phr, 4 phr, and 6 phr resin powder were filled into the NR, respectively. Then the composites were vulcanized at 143 °C for 40 min.

#### 2.3.4. Micromorphology Analysis

In order to observe the dispersement of the short fibers in the NR matrix, the fiber/NR composite specimens were quenched in liquid nitrogen, and a section of the surface was coated with platinum for 45 s in a sputter coater (Sputter Coater 108, Cressington Scientific Instruments Ltd., Watford, UK). Finally, the fracture surface was observed with a scanning electron microscope (SEM, VEGA 3, Tescan, Czech Republic) at 15 kV. The interface between the fiber and the NR was analyzed to determine the compatibility between the UHMWPE short fiber and the NR.

## 3. Results and Discussion

### 3.1. DSC of UHMWPE Short Fiber

**Figure 1:**
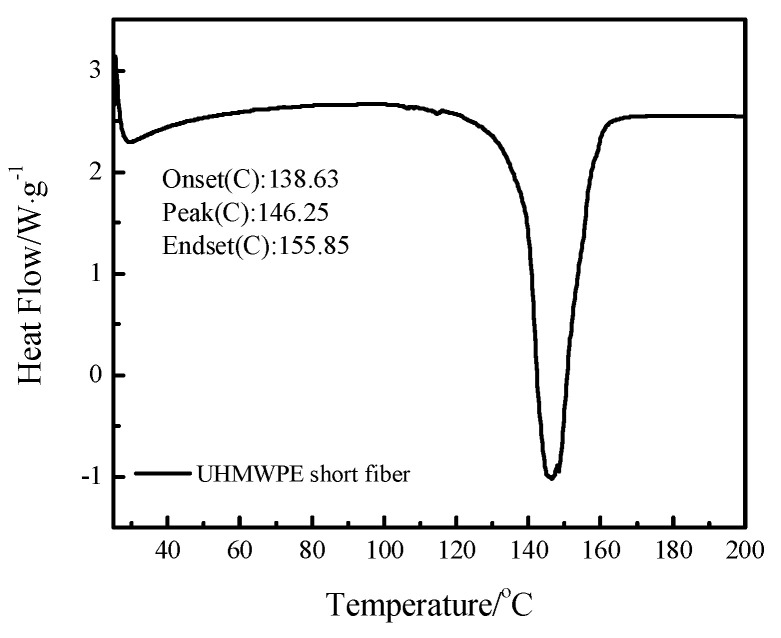
The DSC curves of the UHMWPE short fiber.

The thermal characteristics of the UHMWPE short fiber are shown in Figure 1. It can be seen that the fiber starts melting at approximately 138.6 °C. Furthermore, the melting peak is approximately 146.3 °C; this means that the fiber will lose its excellent mechanical performance once the temperature exceeds 146 °C due to macromolecular disorientation [22]. Thus, the vulcanization temperature of the NR/Fiber composite must be below 146.3 °C and even below 138.6 °C in order to guarantee excellent mechanical performance. However, considering the efficiency of the vulcanization process, three temperatures, e.g., 140 °C, 143 °C, and 150 °C, were still employed as the possible curing temperatures. 

The UHMWPE short fiber has poor heat deformation resistance compared with the aramid short fiber. Cross-linking between the orientated macromolecules of the fiber can improve the resistance to heat deformation. However, simultaneous control of orientation and cross-linking is difficult; cross-linking at a later time, e.g., cross-linking after orientation completion, could provide good resistance to heat deformation or disorientation. Cross-linking by radiation may be competent if the irradiation dosage is not too large. Another later cross-linking may be realized by mixing HDPE being grafted with siloxane before fiber formation and then slowly curing by moisture in the air. A conservative vulcanization temperature (140 °C or 143 °C) for the UHMWPE short fiber with a melting peak at approximately 146.3 °C, compared to 150–160 °C, may provide better results. 

### 3.2. Curing Characteristics of the NR and the UHMWPE Fiber/NR

The curing performances of the rubber compounds are shown in Table 2. These results show that the addition of the fiber had an insignificant effect on the dynamics of the vulcanization. It appears that the vulcanization period can be reduced significantly by filling with CB. However, when TS1 is deduced, it can be seen that the vulcanization periods are not obviously different, and they are all in the 16–23 min range. However, in order to ensure sufficient vulcanization, the optimized vulcanization process temperature was set at 143 °C over 40 min. In order to obtain higher vulcanization efficiency, a temperature of 150 °C over 30 min was also investigated.

### 3.3. Mechanical Properties of the UHMWPE Fiber/NR Composites

Due to the UHMWPE fiber being impressionable to temperature, three different vulcanization temperatures were studied. The fiber/NR composites with different fiber contents were first vulcanized at 140 °C for 40 min to avoid disorientation of the UHMWPE fiber at high temperatures (Table 3). The 2 phr fiber filling was shown to be the best performing, especially with regard to elongation at break. Table 4 lists the mechanical properties of the composites filled with 2 phr fiber vulcanized under different conditions. For samples with the same 2 phr fiber filling, the best vulcanization process had a temperature of 143 °C and a time of 40 min with regards to the tensile stress at 300%, break, and tear strength. Complete vulcanization at temperatures lower than 143 °C may take longer; a temperature of 150 °C results in the disorientation of the fiber. 

The disorientation of the fiber in the composite intrinsically decreases the reinforcing effect of the fibers in the NR. It must be noted that the composite with 2 phr fiber was prepared in a closed mixing machine; it was still pressed into one piece by a two-roll mill. Thus, in the following sample preparation, only a two-roll mill was used. Moreover, the typical vulcanization of rubber products is 150 °C for 10~15 min. A temperature of 143 °C and a 40 min time period are not effective for practical vulcanization. Maybe a longer vulcanization period would bring better mechanical performance. However, formulas matching low-temperature vulcanization, e.g., at 140 °C for 10~15 min, will be conducted in future studies. For example, an accelerator with higher activity or a new vulcanization agent should be used if the temperature is limited blow 150–160 °C. 

**Figure 2:**
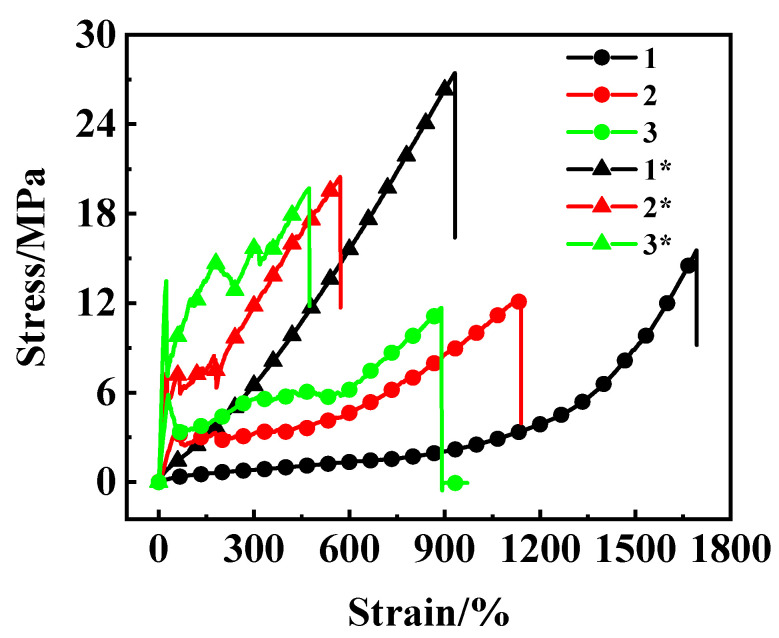
Stress–strain curves of NR and UHMWPE fiber/NR composites. The samples were mixed with the two-roll milling process, and the vulcanization process is 143 °C over 40 min.

The stress–strain curves of the fiber/NR composites are shown in Figure 2. The mechanical properties of the composites are listed in Table 5. The pure NR shows the maximum elongation at break, minimum tensile strength at 300%, and tear strength. The NR/CB samples have greater hardness, tensile strength at 300%, and tear strength compared with the corresponding samples without a CB filling. As the amount of fiber increases, the elongation at break and tensile strength at break both decrease. However, the hardness, tensile stress at 300%, and tear strength all increases significantly. The decrease in elongation and tensile strength at break may be caused by cavitation or disconnection during stretching, which occurs at the interface between the fiber surface and the NR matrix [23]. The lack of a covalent bond and different elasticities between the fiber and the NR is most likely responsible for such cavitation or disconnection. 

The covalent bond between the fiber surface and the NR can be formed by treating the fiber using plasma or corona in order to form the active chemical group, such as hydroxyl substituents. A coupling agent is then grafted with the terminal chemical group, which can take part in the cross-linking reaction of the rubber. In our earlier studies, we treated hydrophilic silica nanoparticles with the same method and enhanced the interaction between the particles and the rubber matrix and the mechanical properties of the reinforced rubber [24,25]. The difference in elasticity between the fiber and the NR can be adjusted by shortening the length of the fiber, i.e., to 1~3 mm. In practical productions, the tear strength of the rubber can be improved using aramid short fiber with a length of 1~3 mm. This area of work will be reported in future studies.

**Figure 3:**
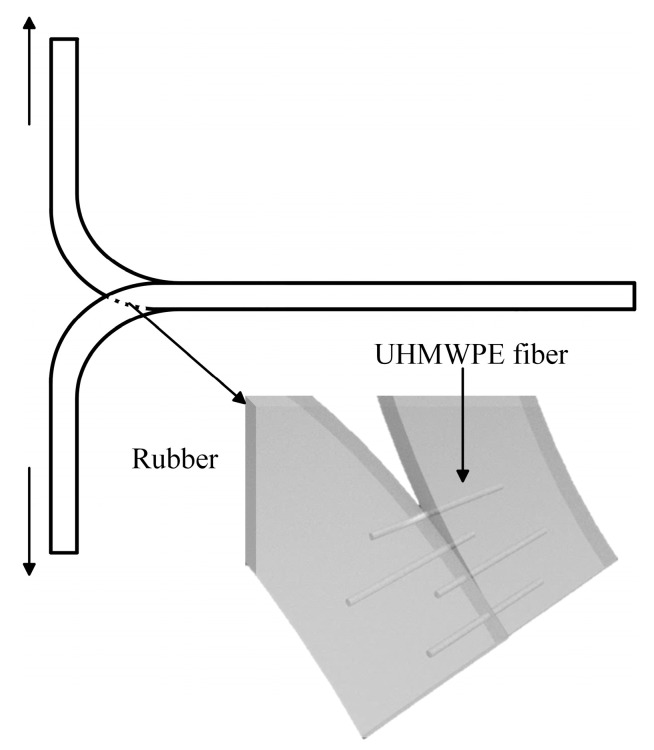
Schematic diagram of the mechanism for improving the tearing strength of NR.

The fibers form a net in the NR matrix when the strain is low; this prevents the expansion of the notch when being torn in a perpendicular direction (Figure 3). This phenomenon indicates that there is significant interfacial interaction between the fiber and the rubber, and so a greater strength is required to pull the fibers out of the rubber matrix. The same surface hydrophobic property of the fiber and the NR is likely responsible for the good interfacial compatibility. At a high strain, the UHMWPE fiber net is damaged due to the differing elasticity or deformability between the NR and the fiber; the delaminated fibers work as stress concentrators, which results in premature failure of the composite [26]. This suggests that the composite introduced in this work may be valuable at a strain of less than 450%. 

In fact, the breaking sound caused by the disconnection of the fiber from the NR matrix could obviously be heard when the composite was stretched too much, e.g., exceeded 450%. Fortunately, many applications of rubber conveyor belts do not require high resistance to strain because the skeleton enhancement layer cannot be stretched too long. It is possible that the cover layer rubber requires high deformability. However, higher elongation at break may be possible by using shorter fibers, i.e., with lengths of 1~3 mm. However, the disconnection of the fiber from the NR matrix at high strain could be avoided by the introduction of a covalent bond between the fiber surface and the NR. In tires and rubber conveyor belts, the steel wire or the textile which are employed to reinforce the rubber are typically treated with copper or adhesion to ensure they can be firmly combined with the NR. 

### 3.4. Dispersement of the UHMWPE Short Fibers in the NR Matrix

**Figure 4:**
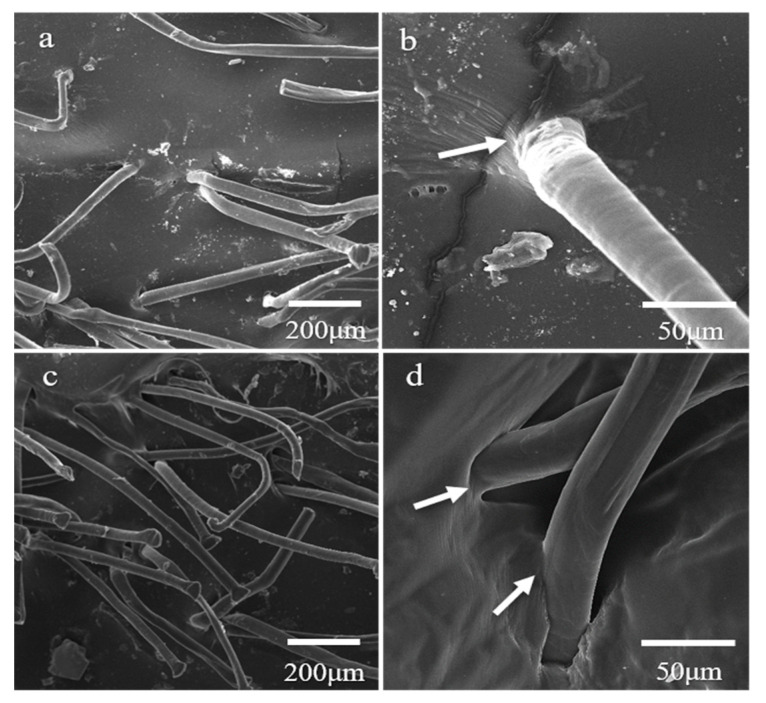
SEM images of the cross-section of the NR filling with 2 phr (**a**,**b**) and 4 phr (**c**,**d**) of UHMWPE short fibers (2 cm length).

Although the naked eye can clearly see that the UHMWPE fibers are homogeneously dispersed on the NR matrix, SEM was still used to observe the interface between the fiber and the NR matrix. The results (Figure 4) show that the fibers are well dispersed in the NR matrix vulcanized at 143 °C for 40 min and that the addition of 4 phr of fiber may be too much. The fibers show no obvious curling and creep behavior; thus, their mechanical properties are not changed significantly. By focusing on each individual fiber, it can be seen that the interface between the fiber and the NR matrix is not sharp (as the arrows show in b,d). This proves that the rubber and UHMWPE molecular chains are entangled together at the micro level.

Anisotropic dispersement of the short fiber in the NR matrix (when all the fibers are orientated along the running direction of the two-roll mill) is obvious when the composite is pressed into a thin piece by two-roll milling. Direct use of the closed mixer can avoid such anisotropy; however, for the formation of the thin piece, two-roll milling and orientation of the fibers are unavoidable. Such anisotropy of the short fiber may be avoided by using a thicker piece, especially when the length of the fiber is shorter. Another strategy for avoiding anisotropy is the perpendicular overlaying of multiple thin pieces. 

### 3.5. NR/UHMWPE Resin Powder Composite 

**Figure 5:**
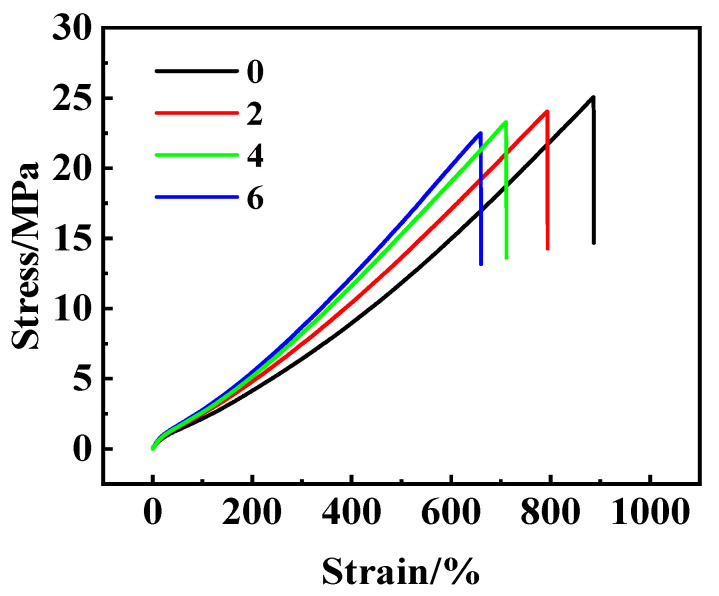
Stress–strain curves of NR/UHMWPE resin powder composites.

The mechanical performance is shown in Figure 5. It can be seen that the trend, which is the same as that shown in Figure 2, is observed, e.g., the higher the content of the resin powder, the lower the strength and elongation at break. Moreover, compared with the NR/fiber composite, the NR/powder composites have a higher elongation at break. This is likely because the applied powder only acts as a type of inert filler and so may not have been stretched into the fiber. This is further evidenced by the tear strengths and the respective changes observed following an increase in powder content (Table 6). Compared with the NR/short fiber composites, the NR/powder composite has a much lower tear strength. 

## 4. Conclusions

In this work, UHMWPE short fibers possessing excellent mechanical properties were dispersed in the NR matrix in order to improve the tear resistance of the NR. Using the two-roll milling process and a suitable vulcanization temperature that is as high as possible but lower than the melting temperature of the UHMWPE (146.3 °C), UHMWPE short fibers/NR composites with significantly enhanced tear resistance and improved tensile stress at 300% elongation, were prepared. Among these composites, the composites consisting of 2 phr fibers exhibit the best comprehensive performance. The tear resistance was improved from 17.1 kN/m to 42.8 kN/m. SEM results indicated that the fibers are well-dispersed and show good interfacial performance with the NR matrix. Although the fiber surface has not been treated, they have natural compatibility with the rubber matrix. This work is expected to provide insight into the industrial preparation of conveyor belts. For example, the use of covalent bonds between the fiber surface and rubber, as well as the use of fibers with a shorter length, will allow for composites with better performance to be prepared.

## Figures and Tables

**Table 1:** Compounding formulation of NR/UHMWPE short fibers.

Sample	NR	CB	Oil	ZnO	SA	Sulfur	TBBS	UHMWPE Short Fiber/Phr
1	100	0	6	5	3	2.2	0.8	0
2	100	0	6	5	3	2.2	0.8	2
3	100	0	6	5	3	2.2	0.8	4
1 *	100	50	6	5	3	2.2	0.8	0
2 *	100	50	6	5	3	2.2	0.8	2
3 *	100	50	6	5	3	2.2	0.8	4

**Table 2:** Vulcanization data of NR/Fiber compounds at 143 °C.

Sample	M_L_/N·m	M_H_/N·m	Ts1/min	T_90_/min
1	0.97	6.41	25.68	44.26
2	0.64	6.84	24.10	42.27
3	0.52	5.98	25.67	45.99
1 *	2.75	20.38	10.61	27.95
2 *	2.50	22.05	9.66	26.20
3 *	2.26	18.45	10.39	33.64

**Table 3:** Mechanical properties of fiber/NR composites with different fiber contents.

Sample	Vulcanization	Tensile Stress at 300% (MPa)	Elongation at Break (%)	Tensile Stress at Break (MPa)	Tear Strength (kN/m)
1 *	140 °C, 40 min	4.97 ± 0.30	1035 ± 31	25.38 ± 0.30	13.05 ± 0.74
2 *	140 °C, 40 min	9.70 ± 0.76	628 ± 23	18.77 ± 0.46	32.41 ± 1.21
3 *	140 °C, 40 min	12.27 ± 1.8	412 ± 71	15.19 ± 0.45	50.36 ± 2.99

Note: * means sample filling with CB, please see Table 1 (the same as following tables or figures).

**Table 4:** Mechanical properties of fiber/NR composites vulcanized under different conditions.

Sample	Vulcanization	Tensile Stress at 300% (MPa)	Elongation at Break (%)	Tensile Stress at Break (MPa)	Tear Strength (kN/m)
2 *	140 °C, 40 min	9.70 ± 0.76	628 ± 23	18.77 ± 0.46	32.41 ± 1.21
2 *	143 °C, 40 min (closed mixing)	10.65 ± 2.2	652 ± 24	18.85 ± 0.83	40.92 ± 2.30
2 *	150 °C, 30 min	9.26 ± 1.2	625 ± 36	20.20 ± 0.55	31.86 ± 2.68

**Table 5:** Mechanical properties of fiber/NR composites with different formulas.

Sample	Hardness (HA)	Tensile Stress at 300% (MPa)	Elongation at Break (%)	Tear Strength (kN/m)
1	39 ± 0.7	0.82 ± 0.03	1680 ± 95	9.5 ± 1.2
2	44 ± 1.2	3.15 ± 0.28	1239 ± 41	14.7 ± 3.5
3	46 ± 1.0	5.25 ± 0.50	891 ± 73	18.6 ± 2.6
1 *	63 ± 0.6	6.49 ± 0.13	912 ± 53	17.1 ± 0.9
2 *	75 ± 0.9	11.42 ± 1.5	576 ± 41	42.8 ± 3.0
3 *	77 ± 0.8	14.64 ± 1.9	484 ± 66	64.9 ± 4.1

**Table 6:** Mechanical properties of NR/UHMWPE resin powder composites.

Sample	Hardness (HA)	Tensile Stress at 300% (MPa)	Elongation at Break (%)	Tensile Stress at Break (MPa)	Tear Strength (kN/m)
0	63 ± 0.6	6.41 ± 0.9	886 ± 13	25.06 ± 1.2	16.9 ± 1.5
2	67 ± 0.7	7.47 ± 1.3	793 ± 21	24.03 ± 0.5	17.5 ± 0.8
4	69 ± 0.3	8.22 ± 1.9	710 ± 19	23.28 ± 0.7	18.8 ± 1.0
6	70 ± 0.5	8.68 ± 1.1	659 ± 14	22.50 ± 1.3	20.5 ± 0.7

Note: Sample 0, 2, 4, 6 means the phr content of the resin powder in the NR.

## Data Availability

Data are contained within the article.

## References

[1] Andrejiova M., Grincova A., Marasova D. (2019). Failure analysis of the rubber-textile conveyor belts using classification models. Eng. Fail. Anal..

[2] Andrejiova M., Grincova A., Marasova D. (2016). Measurement and simulation of impact wear damage to industrial conveyor belts. Wear.

[3] Fedorko G., Molnar V., Grincova A., Dovica M., Toth T., Husakova N., Taraba V., Kelemen M. (2014). Failure analysis of irreversible changes in the construction of rubber-textile conveyor belt damaged by sharp-edge material impact. Eng. Fail. Anal..

[4] Roy K., Debnath S.C., Potiyaraj P. (2020). A critical review on the utilization of various reinforcement modifiers in filled rubber composites. J. Elastomers Plast..

[5] Hashemi S.J., Sadooghi A., Rahmani K., Nokbehrosta S. (2020). Experimental determining the mechanical and stiffness properties of natural rubber FRT triangle elastic joint composite reinforcement by glass fibers and micro/nano particles. Polym. Test..

[6] Tian X.L., Han S., Zhuang Q.X., Bian H.G., Li S.M., Zhang C.Q., Wang C.S., Han W.W. (2020). Surface Modification of Staple Carbon Fiber by Dopamine to Reinforce Natural Latex Composite. Polymers.

[7] Dong C.L., Shi L.C., Li L.Z., Bai X.Q., Yuan C.Q., Tian Y. (2017). Stick-slip behaviours of water lubrication polymer materials under low speed conditions. Tribol. Int..

[8] Pittayavinai P., Thanawan S., Amornsakchai T. (2017). Comparative study of natural rubber and acrylonitrile rubber reinforced with aligned short aramid fiber. Polym. Test..

[9] Roy K., Debnath S.C., Tzounis L., Pongwisuthiruchte A., Potiyaraj P. (2020). Effect of Various Surface Treatments on the Performance of Jute Fibers Filled Natural Rubber (NR) Composites. Polymers.

[10] Moonart U., Utara S. (2019). Effect of surface treatments and filler loading on the properties of hemp fiber/natural rubber composites. Cellulose.

[11] Shen Z., Song W.H., Li X.L., Yang L., Wang C.Y., Hao Z., Luo Z. (2021). Enhancing performances of hemp fiber/natural rubber composites via polyhydric hyperbranched polyester. J. Polym. Eng..

[12] Sathi S.G., Jeon J., Kim H.H., Nah C. (2019). Mechanical, morphological and thermal properties of short carbon and aramid fibres-filled bromo-isobutylene-isoprene rubber vulcanised with 4, 4′ bis(maleimido)diphenylmethane. Plast. Rubber Compos..

[13] Lin G.Y., Wang H., Yu B.Q., Qu G.K., Chen S.W., Kuang T.R., Yu K.B., Liang Z.N. (2020). Combined treatments of fiber surface etching/silane-coupling for enhanced mechanical strength of aramid fiber-reinforced rubber blends. Mater. Chem. Phys..

[14] Chhetri S., Bougherara H. (2021). A comprehensive review on surface modification of UHMWPE fiber and interfacial properties. Compos. Part A Appl. Sci. Manuf..

[15] Li W.W., Li R.P., Li C.Y., Chen Z.R., Zhang L. (2017). Mechanical Properties of Surface-Modified Ultra-High Molecular Weight Polyethylene Fiber Reinforced Natural Rubber Composites. Polym. Compos..

[16] Wang L., Gao S.B., Wang J.J., Wang W.C., Zhang L.Q., Tian M. (2018). Surface modification of UHMWPE fibers by ozone treatment and UV grafting for adhesion improvement. J. Adhes..

[17] Fang Z.H., Tu Q.Z., Shen X.M., Yang X., Liang K., Pan M., Chen Z.Y. (2022). Biomimetic surface modification of UHMWPE fibers to enhance interfacial adhesion with rubber matrix via constructing polydopamine functionalization platform and then depositing zinc oxide nanoparticles. Surf. Interfaces.

[18] Fang Z.H., Tu Q.Z., Chen Z.Y., Shen X.M., Pan M., Liang K., Yang X. (2022). Study on catechol/tetraethylenepentamine and nano zinc oxide co-modifying ultrahigh molecular weight polyethylene fiber surface to improve interfacial adhesion. Polym. Adv. Technol..

[19] Li W.W., Meng L., Ma R.L. (2016). Effect of surface treatment with potassium permanganate on ultra-high molecular weight polyethylene fiber reinforced natural rubber composites. Polym. Test..

[20] Sa R.N., Wei Z.H., Yan Y., Wang L., Wang W.C., Zhang L.Q., Ning N.Y., Tian M. (2015). Catechol and epoxy functionalized ultrahigh molecular weight polyethylene (UHMWPE) fibers with improved surface activity and interfacial adhesion. Compos. Sci. Technol..

[21] Andideh M., Ghoreishy M.H.R., Soltani S., Sourki F.A. (2021). Surface modification of oxidized carbon fibers by grafting bis (triethoxysilylpropyl) tetrasulfide (TESPT) and rubber sizing agent: Application to short carbon fibers/SBR composites. Compos. Part A Appl. Sci. Manuf..

[22] Zhong F., Schwabe J., Hofmann D., Meier J., Thomann R., Enders M., Mulhaupt R. (2018). All-polyethylene composites reinforced via extended-chain UHMWPE nanostructure formation during melt processing. Polymer.

[23] Roy K., Debnath S.C., Das A., Heinrich G., Potiyaraj P. (2018). Exploring the synergistic effect of short jute fiber and nanoclay on the mechanical, dynamic mechanical and thermal properties of natural rubber composites. Polym. Test..

[24] Wang D.L., Chen S., Chen L., Chen B.Y., Ren F.J., Zhu C.X., Feng J. (2019). Investigation and improvement of the scorch behavior of silica-filled solution styrene-butadiene rubber compound. J. Appl. Polym. Sci..

[25] Wang D.L., Ren F.J., Zhu C.X., Feng J., Chen S., Shen G.L., Wang F.F. (2019). Hybrid silane technology in silica-reinforced tread compound. Rubber Chem. Technol..

[26] Meng L., Li W.W., Ma R.L., Huang M.M., Cao Y.B., Wang J.W. (2018). Mechanical properties of rigid polyurethane composites reinforced with surface treated ultrahigh molecular weight polyethylene fibers. Polym. Adv. Technol..

